# EEG “Signs” of Verbal Creative Task Fulfillment with and without Overcoming Self-Induced Stereotypes

**DOI:** 10.3390/bs10010017

**Published:** 2019-12-29

**Authors:** Natalia V. Shemyakina, Zhanna V. Nagornova

**Affiliations:** Sechenov Institute of Evolutionary Physiology and Biochemistry, Russian Academy of Sciences, Toreza, 44, Saint-Petersburg 194223, Russia; nagornova_zh@mail.ru

**Keywords:** creative story generation, EEG spectral power, EEG frequency, mental effort, self-induced stereotypes

## Abstract

The study aimed to reveal task-related differences in story creation with and without the mental effort of overcoming self-induced stereotypes. Eighteen right-handed subjects (19.3 ± 1.1 years old) created stories. The subjects reported the formation of story plot stereotypes (as we call them: self-induced) during self-regulated creative production, which had to be overcome with the instruction to continue the story. Creative task fulfillment (without formed stereotypes—first stage of creation) was characterized by a decrease in the wave percentages of 9–10 Hz, 10–11 Hz and 11–12 Hz frequencies and EEG desynchronization (decreases in EEG spectral power) in the theta (4–8 Hz), alpha1 (8–10 Hz) and alpha2 (10–13 Hz) frequency bands in comparison with the REST (random episodic silent thought) state. The effortful creation task (with overcoming of self-induced stereotypes-second stage of creation) was characterized by increases in waves with frequencies of 9–10 Hz, 10–11 Hz, 11–12 Hz in temporal, occipital areas and pronounced EEG synchronization in alpha1,2 frequency bands in comparison with the free creation condition. It was also found, that the participants with the higher originality scores in psychological tests demonstrated increased percentage of high frequencies (11–12 Hz in comparison with those who had lower originality scores. Obtained results support the role of alpha and theta frequency bands dynamics in creative cognition.

## 1. Introduction

People solve different problems every day. We get used to certain algorithms of actions and their execution becomes automatic. In thinking, we get used to certain schemes, associations that gradually become automatic, stereotypical. Stereotype is defined as a fixed and oversimplified image or idea of something. Stereotypes simplify our life and allow us to make quicker decisions. On the other hand, they can inhibit our creativity [1]. From a broader perspective, not only the perception of something but patterns of behavior and thinking could be called stereotypical. We ourselves can implicitly form the boundaries of our perception of situations or things and form “thinking stereotypes” that can constrict the novelty of our ideas. These types of stereotypes often occur in our daily lives and can disappear if an event begins to contradict them. This is why expanding life experience can increase creativity [2,3]. We called this type of “thinking stereotypes” in the perception of a situation or problem as “self-induced”, “self-created” stereotypes as they seem to be dependent on individual experience.

There are situations when solving a problem narrows an individual’s thinking through self-induced stereotypes and prevents the possibility of producing novel decisions and building a point of view of a situation. These stereotypes could be eliminated by a novel experience or exposure to the ideas of the other in brainstorming [4]. The influence of such stereotypes can be overcome by one on his own by making some effort to consider the situation (or problem) from different sides. It is expected that overcoming a self-created stereotype demands mental effort. Mental effort and any engagement in task fulfillment have classically been connected to a reduction in alpha spectral power [5,6,7]. The greater the alpha frequency reduction is, the greater the mental effort/task demand and arousal associated with an explored task are [8]. It has also been described that a reduction in alpha spectral power is connected to mental load, task difficulty [7,9] and increased attentional performance [10]. The opposite process, that is, an increase in alpha power, may be related to an inhibitory top-down control of executive functioning [11] and the inhibition of task-irrelevant neural circuits [6,12]. In the context of investigations of creativity, an increase in synchronization in the EEG (electroencephalographic) alpha band, interpreted as a decrease in the external attention level, has been discussed as a marker of the incubation stage during essay creation in highly creative subjects [13]. The effects of an alpha spectral power increase connected with shifts in attentional focus have been discussed in several studies [14,15] as a phenomenon of inner attention during verbal creative task performance also [16,17,18]. Thus, as very different effects have been described for mental effort during the performance of different tasks and successful creative task performance (with possible attribution to some “creative effort”), it should be determined what the EEG effects are on free “creative” mental activity and on the effort involved in overcoming self-created stereotypes during verbal creative task fulfillment. 

“Standardly”, creativity is defined as the ability to produce something novel and useful [19]. We also consider creativity to be the ability to produce novel ideas in both open, divergent conditions and in conditions of overcoming a stereotype.

This study aimed to explore rearrangements in EEG frequency percentages (as suggested in [20]) and EEG spectral power changes during creative task performance, with and without mental effort, while overcoming self-induced stereotypes. We wanted to determine what is crucial for creative task performance and for overcoming self-created stereotypes. We expected that the overcoming of self-induced stereotypes might be a special state in creative thinking that should demand an increase in top-down regulation. 

## 2. Materials and Methods

### 2.1. Participants

Eighteen right-handed [21] subjects (mean age 19.3 ± 1.1 years (SD), seven men) took part in the investigation. All procedures were carried out in accordance with the Declaration of Helsinki (1974) and its updates. The protocol of the investigation was approved by the Biomedical Ethics Committee of IEPHB RAS—1/04 (April 2018), project identification code—PSYPHYS01-2018. Participants reported no psychiatric, psychological, substance use or dependence disorders, and were not taking any current medications. All subjects signed written consent forms before participating in the study. 

### 2.2. Psychological Testing

IQ was assessed using the standard progressive matrices [22]. The matrices test is a non-verbal, culturally independent IQ test that measures deductive reasoning through five different sets of multi choice tasks. The “multiple uses test” and “generation of sentences” test were used to measure the creativity potential of each subject. In the latter test, subjects were required to compose as many sentences as they could during a limited period of time after being given the first letter of the words. For both tests, assessments were performed for originality, fluency, and flexibility in accordance with the original scoring procedure [23]. 

### 2.3. Tasks in the EEG Study

The stimuli used for story creation during EEG registration were pictures from the Guilford and O’Sullivan social IQ Test [24]. We used black-and-white pictures with only two personages. 

Free creation task (FCrT). Subjects were told that what they could see in the picture was the middle of a situation. They had to create the plot before and after it. Subjects were asked to mentally think in sentences while creating the story and to voluntarily press a button when they were ready to tell the story. They were given approximately 2–3 min to produce the story. 

Effortful creation task (EffCrT). After the subjects had already told the plot of the story, they were asked to continue the story they had just created and were to mentally produce new changes in its plot using the same picture presentation. They also had to voluntarily press the button when they were ready with the new original followings of the story and were asked to tell the plot changes.

In both cases, subjects gave feedback about the stories that they have created, namely, on the task’s difficulty, according to a point scale of 1–10 (1—easy; 10 —not possible to do), and the emotional state (–3; +3, where “–3” was the most negative state, “+3”—the most positive, and 0 was neutral) connected with the fulfillment of the task. To eliminate the novelty effects on the EEG, subjects had to fulfill the story creation training task but without the part of overcoming a stereotype (3–6 min) before the EEG investigation. The order of the pictures used for the subjects’ story creations was randomized among the subjects. 

**Figure 1:**
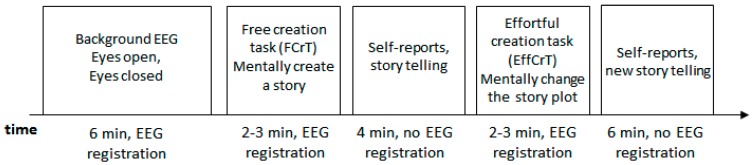
The timeline of the EEG recording.

The timeline of the investigation procedure is depicted on Figure 1. 

### 2.4. The Procedure, EEG Data Acquisition, and Analysis

Participants were tested individually in a single experimental session. The electroencephalograms (EEGs) were recorded using a Mitsar 32-channel EEG system (Mitsar, Ltd. St. Petersburg, http://www.mitsar-medical.com) by means of the WinEEG software package (Ponomarev V.A, Kropotov Ju.D. The RF register for the computer programs #2001610516, 08.05.2001). AgCl electrodes were positioned according to the modified 10–10 system (Fp1, Fpz, Fp2, F7, F3, Fz, F4, F8, T3, C3, Cz, C4, T4, T5, P3, Pz, P4, T6, O1, Oz, and O2). The input signals were referenced to the linked ears, were filtered between 0.53 Hz and 30 Hz and digitized at a rate of 500 Hz. The ground electrode was located nearby Fpz site. The resistance of the electrodes did not exceed 5 ohms. 

*Artifact rejection.* Eye-blink artifacts were corrected by zeroing the activation curves of the individual independent components corresponding to eye blinks. These components were obtained via application of independent component analysis (ICA) of the raw EEG fragments (described in [25]). High- and low-frequency activities were marked as artifacts and were excluded from further analysis. The thresholds were set as follows: (1) 50 µV for slow waves in the 0–2 Hz band and (2) 35 µV for fast waves in the 20–35 Hz band.

*Spectral power analysis.* Calculation of the EEG spectral power was performed using a standard approach [26]. For each individual, each observation and each EEG channel power spectra were computed separately, as follows. The continuous EEG data were segmented into 4.096 s epochs (50% overlap). All epochs containing artifacts were removed from the analysis. The spectral power was computed using a Hanning window and fast Fourier transform (FFT) and was averaged across epochs. A number of averaged non-artifact epochs for the power spectra analysis were balanced within the subjects and were set to 45. The estimations of the absolute power of the EEG were calculated and averaged in the following frequency bands: delta (1.5–4 Hz), theta (4–7.5 Hz), alpha1 (7.5–9.5 Hz), alpha2 (9.5–12.5 Hz), beta1 (12.5–18 Hz) and beta2 (18–30 Hz). Estimation arrays were averaged for each subject in each year and were subjected to standardization by means of transformations, Y = log X for power [27], for further statistical analysis. The individual alpha peak frequencies (IAF) were determined visually as the peak of spectral power of EEG in resting state with closed eyes within the alpha band at parietal electrode Pz. 

*EEG frequency structure analysis.* Since the spectral power of EEG rhythms is influenced not only by the index of these rhythms, but also by the amplitude—consideration of the frequency structure and spectral power of EEG gives a broader perspective to the estimation of the neurophysiological mechanisms of cognitive activities and individual differences. For the frequency structure evaluation, the interval durations were measured between the points crossing the zero (isoline) using a curve by means of S.S. Bekshaev’s (IEPhB RAS) program [20,28]. The interval durations (ms) between points on the isoline intersections were transformed into frequencies (Hz) and evaluated across all analyzed EEG intervals (no less than 3 min). The average duration of the analyzed non-artifact time intervals was 2.7 ± 0.3(SD) min per subject. The representation of the rhythmic components (averaged with 1 Hz step) was expressed as the percentage of waves for each frequency relative to the total number of registered waves (based on zero crossing). The analysis of the frequency structure of the EEG was carried out for all the electrode sites. 

*Statistical analysis.* The differences in spectral power were estimated for each frequency band (theta, alpha1,2, beta1,2) by means of repeated measures ANOVA, with the within-subject factors “TASK” (2 tasks and resting state) and “ZONE” (21 electrode positions) and the between-subject factor “GROUP” (groups with high and average originality). 

EEG frequency changes in each frequency band during task fulfillment were analyzed by means of repeated measures ANOVA considering the within-subject factors TASK (free creation, effortful creation, REST) and ZONE (21 electrode positions) and the between-subject factor GROUP (groups with high and average originality). The results were considered significant with the Huynh-Feldt correction [29]. The alpha level was set to 5%. As the distributions of individual alpha peak frequency did not differ from normal distribution (Shapiro-Wilk test W = 0.94, p = 0.3) between group comparison was performed by t-test. 

## 3. Results

### 3.1. Psychological Results

All subjects had normal IQ scores: 110 ± 8 (SD). The psychological testing revealed inverse correlations between the self-report scores for two verbal creative task difficulties (multiple using test—boxes; creation of sentences using first letters) and the measured originality. They were Rs = −0.6, t(N-2) = −3.1, p < 0.008 and Rs = −0.67, t(N-2) = −3.6, p < 0.002 correspondingly. Based on the summarized originality scores during the fulfillment of the two verbal tasks, the whole group was divided into two groups, namely, more (10 people) and less (eight people) original groups. The originality scores in high originality group were between 10 and 15 points compares to the less “original” group with scores between two and eight points. At the same time being more or less original was not connected with IQ. No group differences were observed between the more and less creative subjects in terms of IQ scores (p < 0.1 by Mann-Whitney test). 

The subjects’ reports suggested the formation of self-induced story plot stereotypes during the self-regulated (free) creative production (first instruction), which was difficult to overcome in the additional time period. We refer a self-induced story plot stereotype as the perception of the pictured situation and characters of the created story in a rigid unchangeable way that may inhibit the further novel creative development of story. 

According to the self-reports the median value of the emotions level was +1 in both tasks (in the “free creation task” the 25–85 quartile were 1–2, in the “effortful creation task” the 25–85 quartile were 0–2). The median value of the difficulty was “four” in the “free creation task” (the 25–85 quartile was 2–6) and “five” in the “effortful creation task” (the 25–85 quartile was 4–7). At the same time there were no significant differences for emotions or difficulty scores in the mentioned tasks, so we consider these tasks as balanced ones by subjective estimations.

### 3.2. EEG Data Analysis

#### 3.2.1. EEG Spectral Power Changes

**Figure 2:**
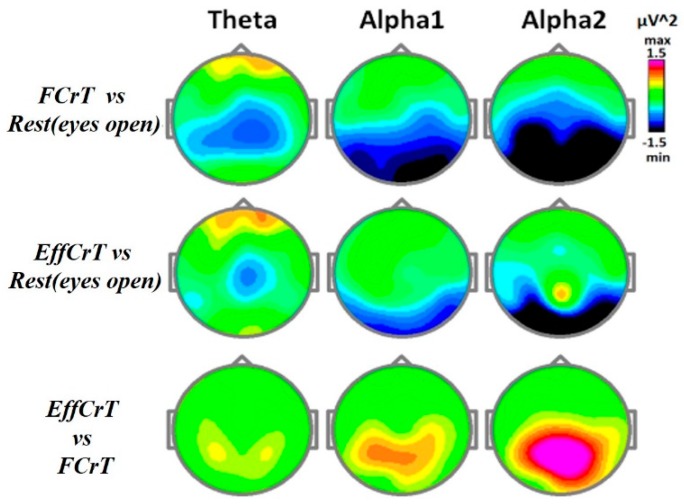
Topographical maps of the spectral power changes during “free creation task” fulfillment and “effortful creation task” fulfillment. Notes: The “free creation task”(FCrT) was the first stage of the task, which was the condition of freely creative activity (Instruction № 1). The “effortful creation task”(EffCrT) was the condition in which the “thinking” stereotype of story plot expected to be overcome (instruction No 2). The vertical bar represents the spectral power differences (squared µV) between the conditions (first task versus the second one) in the theta, alpha1, and alpha2 frequency bands.

A desynchronization effect on the EEG was observed during free and effortful creative task fulfillment, but the more effortful creative task (expected overcoming the self-induced stereotype) demanded a less activated state. Significant differences in the spectral power for the TASK x ZONE interaction were obtained in the theta (F(40, 680) = 3.3, e(H-F) = 0.23 p < 0.00001), alpha1 (alpha1 F(40, 680) = 2.5, e(H-F) = 0.35, p < 0.0002) and alpha2 (alpha2 F(40, 680) = 3.06, e(H-F) = 0.25, p < 0.0001) frequency bands. The topographical details regarding the effects of the spectral power changes on the different frequency bands are presented in Figure 2.

The individual alpha peak frequency (IAF) did not differ between groups with high and “average” originality score (p = 0.4 by T-test). In the whole group (combining more and less original subjects), the mean IAF was 10.5 ± 0.6 Hz. 

#### 3.2.2. EEG Frequency Changes

Between tasks, the EEG frequency percentage differences were obtained only for three frequencies (9–10 Hz, 10–11 Hz, 11–12 Hz)—upper borders of the low alpha and high alpha. Significant effects of the factor TASK were revealed on the frequencies from 9 to 10 Hz (F(2,32) = 11.0, e(H-F) = 0.9, p = 0.0004) and from 10 to 11 Hz (F(2,32) = 11.5, e(H-F) = 0.8, p = 0.0005). The effects of the TASKxZONE interaction were revealed on the 9–10 Hz frequency (F(40,640) = 1.7, e(H-F) = 0.5, p = 0.03) and on the 11–12 Hz frequency (F(40, 640) = 1.6, e(H-F) = 0.6, p = 0.04). 

**Figure 3:**
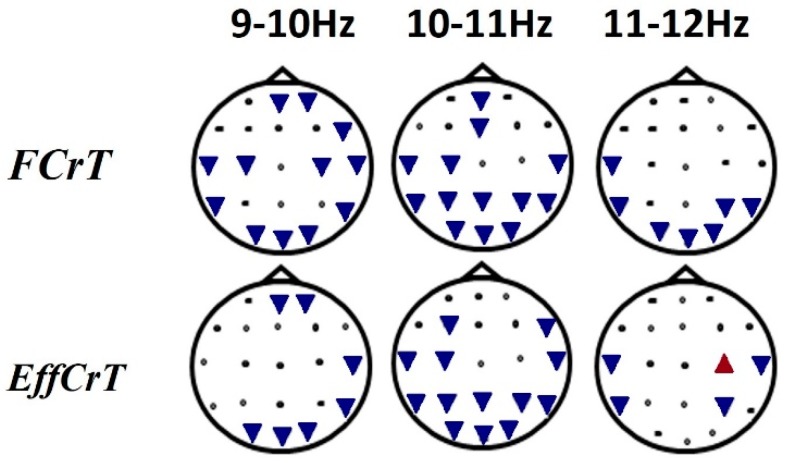
Frequency percentage differences during task fulfillment versus the resting-state condition. Notes: The “free creation task”(FCrT) was the first stage of the task, which was the condition of freely creative activity (instruction No 1), The “effortful creation task”(EffCrT) was the condition in which the thinking stereotype about the story plot expected to be overcome (instruction No 2). The triangles directed toward the bottom of the corresponding electrode position indicates a lesser percentage of the waves in the corresponding frequency bands during task fulfillment versus the resting state. The triangles directed toward the top of the corresponding electrode position indicate a greater percentage of the waves in the corresponding frequency bands during task fulfillment versus the resting state.

During creative task fulfillment, the percentage of the frequencies from 9 to 12 Hz was lower than that in the resting state (Figure 3).

#### 3.2.3. EEG Frequency Differences between the High and Low Creative Originality Score Groups

**Figure 4:**
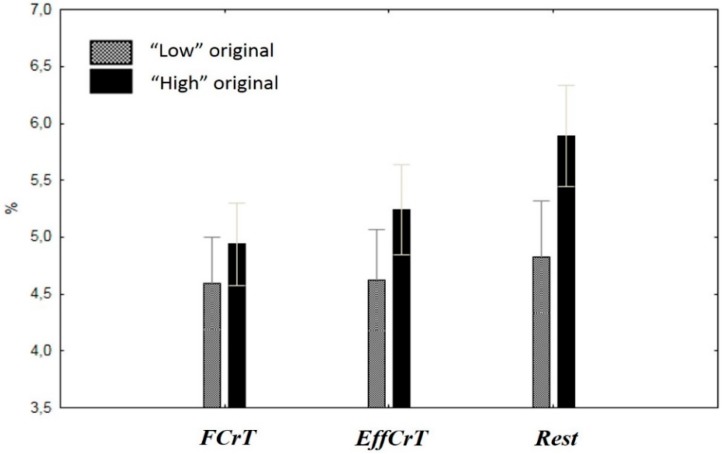
Between-group differences in terms of the 9–10Hz frequency percentage during task fulfillment. Notes: The task indices are on the x-axis, and the values of the 9–10Hz frequency percentage are on the y-axis. Black columns represent the high originality group; gray columns represent the low (average) originality group.

The effect of the interaction of the between-subject factor GROUP and the factor TASK was revealed in the 9–10-Hz frequency (F(2, 32) = 3.9, e(H-F) = 0.6, p < 0.05). Frequency changes between the TASK and REST conditions in the group with higher originality scores were more pronounced than those in the low originality group (Figure 4).

At the same time, the main effect of the between-subject factor GROUP was revealed in the 11–12 Hz frequency (F(1, 16) = 4.6, p = 0.047) related to a higher percentage of the 11–12 Hz frequency in the group with higher originality scores compared with the group with low originality scores in all conditions, namely, the fulfillment of the creative tasks and the rest condition.

Consideration of the spectral power group differences in the wide EEG frequency bands did not reveal significant differences between the groups.

## 4. Discussion

### 4.1. Frequency and EEG Spectral Power Changes during Creative Tasks Fulfillment with and without Overcoming Self-induced Stereotypes

By means of EEG spectral power analysis, both types of creative tasks were characterized by desynchronization effects relative to the REST that is normally reflective of the engagement in the task (for review see [30]). Exploring the frequency structures and the changes in the frequency percentages in the narrow frequency bands (with 1 Hz steps) in the creative conditions, we also observed decreases in the percentages of the alpha frequencies (from 9 to 12 Hz) in comparison with the REST state. In [31,32] was observed a decrease in EEG power in the alpha1 and alpha2 frequency bands during the completion of verbal creative tasks compared to background EEG. In the research of [33], EEG desynchronization in the alpha band (7.5–12.5 Hz) was observed during the alternate uses task and a word association task compared to the prestimulus reference EEG intervals. Thus, our results demonstrated unidirectional changes both in the spectral power of the wide frequency bands and in the percentage of alpha frequencies within the EEG frequency structure during creative task fulfillment in comparison with REST.

At the same time, the “effortful creation” task revealed an increase in EEG power in the theta and the low and high alpha bands compared to the “free creation” condition. These differences were more pronounced in the high alpha band in the parietal cortex areas. In the study by [34], when comparing the performance of a verbal creative task (essay writing) with less creative and non-creative tasks, an increase was observed in the power of the alpha band (alpha-2, 10.3–12.3 Hz). The increases in the alpha spectral power might be interpreted as one of the signs of creative activity [33,35]. There are several assumptions regarding the physiological cause of such increases.

Increases in the power of the alpha frequency bands from one hand can negatively correlate with the perception of the presented stimuli [36,37,38,39], demonstrate less activated states [40], and exhibit a suppression of visual information distractions [41,42] in conditions exploring the relationship between alpha rhythms and attentional shifts. Thus, an increase in alpha wave activity may be associated with the filtering of external information through the regulation of the level of excitation and inhibition in the cortex as a result of a top-down controlling influence [11]. The increase in the EEG power in the alpha band during verbal creativity might be related to the redistribution of the focus of attention inward and the cessation of external sensory stimulation for the successful fulfillment of a creative task and the associative search. In the studies of several psychologists [43,44,45], defocused attention was marked as the necessary condition for the enlarged borders of a successful associative search (for review see, for example [46]). Physiological investigations of creativity have demonstrated an increase in the alpha frequency power in both the prefrontal and parietal cortex in different conditions in which creative ideas appearing [33,35,47]. In another work, event-related frontal alpha synchronization was revealed in both divergent (generation of sentences with a given letter) and convergent (anagrams solution) thinking tasks in response to high internal processing demands but not in response to low internal processing demands when bottom-up processing was allowed. The authors connected the frontal alpha synchronization to the high internal processing demands and exclusive top-down control [48]. In our case, we demonstrated an increase in the alpha frequency power in the associative parietal cortex with more pronunciation in the high alpha band for the “effortful creation” task with self-induced stereotypes being overcome in comparison with the “free creation” task. The observed effects corresponded to an increase in the creative “effort” during the task that involved overcoming self-induced stereotypes, as significant differences between tasks were also observed in connection with the slight increase in theta power that also occurred in the parietal cortex, which could be considered associative memory engagement [49,50,51,52]. The data in the literature have demonstrated an increase in theta activity in the optimal conditions for stabilization of new hippocampus-dependent memories [53].

Many investigations have demonstrated tightly bound alpha band changes with creativity effectiveness. It was also shown that stimulation of creative thinking was associated with an increase in the power of the alpha-2 (10–12 Hz) frequency band [54]. Over the last several years, with the growing data on stimulation investigations, it has been shown that tACS (transcranial Alternating Current Stimulation) at 10 Hz on frontal cortex zones increases the results of creative activity, while the more expected higher stimulation in the gamma range (40 Hz) does not produce such an effect [55]. That serves to understand the special role of alpha oscillations in creative thinking.

The redistribution of the attention focus inward and the inhibition of external sensory stimulation (an increase in alpha power) for the successful fulfillment of a creative problem [16,17] is something that could be crucial for overcoming a stereotype for more creative decisions. An attempt to overcome self-formed stereotypes should demand an increase in top-down regulation and attentional rearrangements; our hypothesis was based on a series of studies in which, using various methodological techniques, it was found that the increase in the power of the high alpha band (alpha2, 10–13 Hz) in the posterior cortex a reduced excitation of cortical areas in response to visual stimulation. However, it also should be mentioned, that there was observed such an effect for self-induced short-term memory stereotypes and did not clearly observe the same effect in overcoming long-term memory stereotypes [56,57], in that the alpha frequency bands were not sensitive. Most of the results were obtained in the beta frequency bands. This observation is very interesting and may be related to the task specificity in both investigations: the ability to overcome long-term memory stereotypes was based on the creation of new endings to the well-known proverbs in the time-limited condition. The time limits (short trials of only several seconds for each decision) led to the necessity for focused attention, high-speed processes and higher working memory load that could be accompanied by increases in beta activity [58,59,60]. In the condition of the absence of a long-term memory stereotypes during the creation of original definitions of words from different semantic fields, decreases in EEG spectral power in the beta-2 frequency band were observed in frontal areas, and an external induction of positive emotions led to generalized increases in the power of the beta-2 band [61]. These findings demonstrated the sensitivity of the high-frequency EEG bands to conditions of creative thinking and flexible changes in states depending on the task demands. Spectral power in the high alpha band while overcoming long-term memory stereotypes (proverbs ending creation) increased slightly in frontal and temporal regions and decreased in parietal regions that corresponded to top-down processes and activation in multisensory parietal areas [56], which is also connected to cognitive processes such as memory requirements and information processing [62].

In the “effortful creation” task, the participants had no time limits and created different changes in the story plot in the condition of the intensive search of the associated field and recombination of semantic information. Synchronization of the upper alpha band in this case, might be connected to the inhibition of the just created mental stereotype (suppression of the initially appearing associations, that are playing the role of stereotypes) and flexible switching from one possible storyline to another. This state of free associations without time limits might be compared with the emergence of a dreaminess state with an internal focus of attention, which can lead to increases in the alpha waves of frequency structure and in the power of the alpha frequency bands.

Therefore, the state of creative effort in overcoming stereotypes is characterized by EEG parameters that are clearly distinct from mental effort and tiredness. The main physiological signs of creative effort in time unlimited conditions are the increases in high alpha EEG spectral power in parietal areas and increases in the frequency of 9–10 Hz, 10–11 Hz and 11–12 Hz percentages in temporal and occipital areas.

### 4.2. Comparing Results in Groups with Lower and Higher Scores of the Originality

The spectral power analysis revealed between-task differences in the wide frequency bands and was not sensitive to the between-group differences, while the frequency structure analysis gave us an opportunity to speculate on the functional role of narrow frequency ranges within the alpha bands during creative ideation. It was shown (Figure 3) that 9–10 frequency changes between tasks and rest conditions in the group with higher originality scores were more pronounced in comparison with the less original group. This finding corresponded to the assumption of Martindale regarding more varied cortical activation patterns and stimuli reactiveness in more creative individuals [63]. In this study, the fulfillment of creative and intellectual tasks differed in terms of the alpha waves percentage in more creative subjects and was similar in less creative persons [64]. We observed a greater decrease in the percentage of the waves with frequencies of 9–10 Hz in creative individuals compared to low creative ones in task performance. In our study, the findings were related to the narrow frequency band (9–10 Hz); thus, the effect could be attributed to greater reactivity in activation in response to the task demand. Therefore, the slow alpha might be an index of cortical activation levels and engagement in task fulfillment. It was shown that participants with intensive creativity training and creative abilities (actors) are characterized by more pronounced EEG changes during the mental performance of emotional, personal and scenic situations than participants without special training or skills (non-actors) [65]. In the study of the autonomic markers of divergent thinking, it was demonstrated that people with high creative achievement (measured by the Creative Achievement Questionnaire) showed significantly greater increases in sympathetic activity between the baseline and the task, reflecting higher effort than in those with low creative achievement [66]. Therefore, the greater decreases (greater reactivity) of the 9–10 Hz frequency in the high creative group during tasks fulfillment reflected the flexibility of their functional state compared to the low original group.

The percentage of 11–12 Hz in the high originality group was higher despite the task type, including the REST state in comparison with the low originality group. The data correspond to those of previous investigations [32,67], wherein enhanced alpha power was more pronounced in highly creative subjects during creative task performance. When comparing the verbal creative activity between high and low creative participants [32], obtained high power values in the alpha1 frequency band and high power values in the alpha2 band in the front and the parietal cortex in the group of more creative participants. The increases in the EEG power of the alpha frequency band was more pronounced in high creative individuals in the creation of more original ideas and the fulfillment of more difficult tasks [67]. In the study by [68], it was discussed that a more pronounced upper alpha synchronization at rest (or during the reference interval) is positively associated with semantic memory performance. According to the alertness hypothesis, the higher alpha power during reference interval signifies greater readiness of the alpha system in information processing [69]. Our results allowed us to confirm the assumption of greater sensitivity and reactivity of creative persons to task demands. Considering the data regarding an increase in the EEG spectral power of the high alpha band at an “effortful creation” task, we can assume an easier transition of the high-original group of participants to the state-promoting associative search.

### 4.3. Study Limitations and Future Research

The main limitation of this study was a small group of participants, that allows to consider only the main effect of the given instructions (in free and effortful creativity tasks). In further research it may be necessary to consider the influence of individual factors such as (i) the success of overcoming the stereotype during creative task performance (assessed both by self-reports and experts evaluations), (ii) influence of personality and thinking style on neurophysiological correlates of creative activity, and (iii) assessing the positive influence of stereotypes (the creative stereotype effect) on creative activities when creating stories. All these issues can be considered with increasing the group of subjects and modeling the conditions of creative activity.

## 5. Conclusions

Among others, we supported the special role of alpha and theta frequency bands for creative task performance and obtained, that it might be crucial for conditions of overcoming self-induced stereotypes. The increasing of the alpha EEG spectral power in “effortful creation” in comparison with the “free creation” condition might be related to external information inhibition and may be important for associative processes and the recombination of semantic information for overcoming self-induced thinking stereotypes, rearrange previous conception and create novel interpretations.

Obtained changes in the 9–10 Hz frequency percentage between the tasks and rest conditions suggest a greater reactivity and more flexible states regulation according to the tasks demands in the group with higher originality scores than in the less original group.

In the high original group, the percentage of 11–12 Hz in EEGs was greater in spite of the task type including a REST state that was not related to the alpha peak frequency. The data corresponded with investigations [32,67], where enhanced alpha power was more pronounced in highly creative subjects during creative task performance. Thus, obtained data supports that overcoming of self-induced stereotypes forms a special state that might be considered as a special type of creative activity.
